# Ethyl 3-[(6-chloro­pyridin-3-yl)meth­yl]-2-oxoimidazolidine-1-carboxyl­ate

**DOI:** 10.1107/S1600536812001948

**Published:** 2012-01-21

**Authors:** Kamini Kapoor, Vivek K. Gupta, Madhukar B. Deshmukh, Chetan S. Shripanavar, Rajni Kant

**Affiliations:** a X-ray Crystallography Laboratory, Post-Graduate Department of Physics & Electronics, University of Jammu, Jammu Tawi 180 006, India; b Department of Chemistry, Shivaji University, Kolhapur 416 004, India

## Abstract

In the title compound, C_12_H_14_ClN_3_O_3_, the imidazole ring adopts a half-chair conformation. The dihedral angle between the pyridine and imidazole rings is 70.0 (1)°. In the crystal, the molecules are linked by C—H⋯O inter­actions, forming chains parallel to the *c* axis.

## Related literature

For background to the insecticidal applications of imidacloprid [systematic name: *N*-[1-[(6-chloro-3-pyrid­yl)meth­yl]-4,5-dihydro­imidazol-2-yl]nitramide], see: Samaritoni *et al.* (2003 ▶); Kagabu *et al.* (1997 ▶, 2007 ▶); Zhao *et al.* (2010 ▶). For ring conformations, see: Duax & Norton (1975 ▶). For related structures, see: Kapoor *et al.* (2011 ▶); Kant *et al.* (2012 ▶).
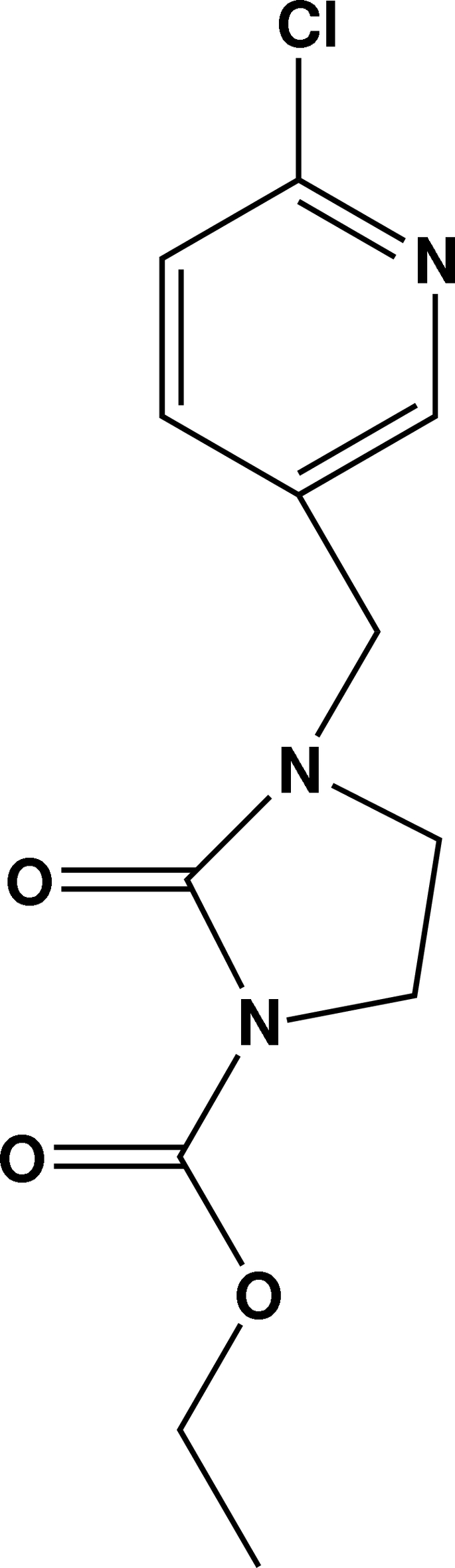


## Experimental

### 

#### Crystal data

C_12_H_14_ClN_3_O_3_
*M* *_r_* = 283.71
Monoclinic, 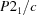

*a* = 13.3926 (14) Å
*b* = 8.4991 (8) Å
*c* = 12.3361 (12) Å
β = 106.538 (10)°
*V* = 1346.1 (2) Å^3^
*Z* = 4
Mo *K*α radiation
μ = 0.29 mm^−1^
*T* = 293 K
0.3 × 0.2 × 0.2 mm

#### Data collection

Oxford Diffraction Xcalibur Sapphire3 diffractometer
Absorption correction: multi-scan (*CrysAlis PRO*; Oxford Diffraction, 2010 ▶) *T*_min_ = 0.941, *T*_max_ = 1.000
6112 measured reflections
2645 independent reflections
1537 reflections with *I* > 2σ(*I*)
*R*_int_ = 0.035

#### Refinement

*R*[*F* ^2^ > 2σ(*F* ^2^)] = 0.065
*wR*(*F* ^2^) = 0.191
*S* = 1.05
2645 reflections
173 parameters
1 restraint
H-atom parameters constrained
Δρ_max_ = 0.42 e Å^−3^
Δρ_min_ = −0.21 e Å^−3^

### 

Data collection: *CrysAlis PRO* (Oxford Diffraction, 2010 ▶); cell refinement: *CrysAlis PRO*; data reduction: *CrysAlis RED*; program(s) used to solve structure: *SHELXS97* (Sheldrick, 2008 ▶); program(s) used to refine structure: *SHELXL97* (Sheldrick, 2008 ▶); molecular graphics: *ORTEP-3* (Farrugia, 1997 ▶); software used to prepare material for publication: *PLATON* (Spek, 2009 ▶).

## Supplementary Material

Crystal structure: contains datablock(s) I, global. DOI: 10.1107/S1600536812001948/gg2073sup1.cif

Structure factors: contains datablock(s) I. DOI: 10.1107/S1600536812001948/gg2073Isup2.hkl

Supplementary material file. DOI: 10.1107/S1600536812001948/gg2073Isup3.cml

Additional supplementary materials:  crystallographic information; 3D view; checkCIF report

## Figures and Tables

**Table 1:** Hydrogen-bond geometry (Å, °)

*D*—H⋯*A*	*D*—H	H⋯*A*	*D*⋯*A*	*D*—H⋯*A*
C4—H4⋯O1^i^	0.93	2.53	3.366 (5)	150
C9—H9*B*⋯O2^i^	0.97	2.50	3.395 (4)	152

Symmetry code: (i) 
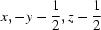
.

**Fig. 1.**
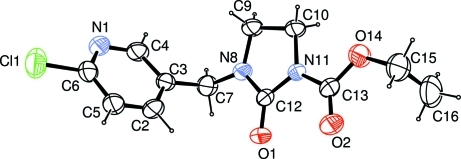
ORTEP view of the molecule with the atom-labeling scheme. The thermal ellipsoids are drawn at the 40% probability level. H atoms are shown as small spheres of arbitrary radii.

**Fig. 2.**
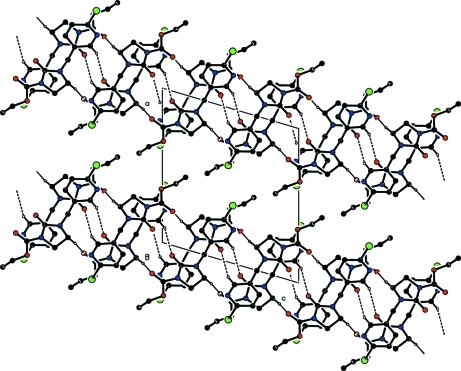
The packing arrangement of molecules viewed in the b axis direction. The dashed lines show the intermolecular C—H···O interactions.

## supplementary crystallographic information

### Comment

Since the advent of imidacloprid, the search for new neonicotinoid insecticides has been intense with competitive efforts by several research groups within the agrochemical industry (Samaritoni *et al.*, 2003). From the crystallographic study of imidacloprid and related insecticides, precise three-dimensional structural information and characteristic molecular features gave an insight of the binding mode of these insecticides to nAChRs (Kagabu *et al.*, 1997). The biological profile of imidacloprid provides an impulse in the development of new products by modifying the structural features of the prototype (Kagabu *et al.*, 2007). Therefore, in a search for new neonicotinoid insecticide with improved profiles, neonicotinoid derivatives containing N-oxalyl groups were designed and synthesized. (Zhao *et al.*, 2010). The bond lengths and angles observed in (I) show normal values and are comparable with related structures (Kapoor *et al.*, 2011; Kant *et al.*, 2012). The imidazole ring adopts a half-chair conformation with asymmetry parameter: ΔC_2_(C9—C10)=1.51 (Duax *et al.*, 1975). The dihedral angle between the pyridine and imidazole rings is 70.0 (1)°. Molecules in the unit cell are packed together to form well defined chainss (Fig. 2). Within the chains, the molecules are linked by two different intermolecular C—H···O interactions (Table 1).

### Experimental

Imidacloprid (10.20 g, 0.04 mol) was dissolved in 30 ml acetone, ethyl chloroformate (6.482 g, 0.06 mol) in the presence of 10 g K_2_CO_3_. The mixture was refluxed for 24 hrs with the progress of the reaction monitored by TLC. After completion of the reaction, the mixture was filtered to remove the K_2_CO_3_, by a process of slow evaporation. White crystalline compound was separated out with 80% yield.

m.p. 362–363 K; IR (KBr) cm-1: 2980, 2903, 1764; ^1^*H*-NMR (300 MHz, CDCl3) δ: 1.28 (t, J=7.5 Hz, CH3), 3.27(t, J=7.5 Hz, CH2), 3.74(t, J=7.5 Hz,CH2), 4.20(q, J=7.5 Hz, OCH2), 4.35(s, CH2), 7.25(d, J=8.2 Hz, py, 1H), 7.61(dd, J=7.5 Hz, J=2.5 Hz, py, 1H), 8.21(s, py, 1H) p.p.m.; ^13^C-NMR (300 MHz, CDCl3) δ: 153.71, 151.72, 151.15, 149.20, 138.93, 130.69, 124.50, 96.05, 62.39, 44.50, 40.52, 14.39 p.p.m.; LCMS/MS (ESI): 284 [*M*+], (m/*z*).240, 256, 212, 172, 126.

### Refinement

All H atoms were positioned geometrically and were treated as riding on their parent C atoms, with C—H distances of 0.93–0.97 Å and with *U*_iso_(H) = 1.2Ueq(C) or 1.5Ueq(methylC). Due to large value of the displacement parameter for C16 and consequent librational motion, the C15—C16 bond length was constrained.

### Crystal data

**Table:**

C_12_H_14_ClN_3_O_3_	*F*(000) = 592
*M**_r_* = 283.71	*D*_x_ = 1.400 Mg m^−^^3^
Monoclinic, *P*2_1_/*c*	Melting point: 362 K
Hall symbol: -P 2ybc	Mo *K*α radiation, λ = 0.71073 Å
*a* = 13.3926 (14) Å	Cell parameters from 1920 reflections
*b* = 8.4991 (8) Å	θ = 3.4–29.0°
*c* = 12.3361 (12) Å	µ = 0.29 mm^−^^1^
β = 106.538 (10)°	*T* = 293 K
*V* = 1346.1 (2) Å^3^	Plate, white
*Z* = 4	0.3 × 0.2 × 0.2 mm

### Data collection

**Table:**

Oxford Diffraction Xcalibur Sapphire3 diffractometer	2645 independent reflections
Radiation source: fine-focus sealed tube	1537 reflections with *I* > 2σ(*I*)
graphite	*R*_int_ = 0.035
Detector resolution: 16.1049 pixels mm^-1^	θ_max_ = 26.0°, θ_min_ = 3.4°
ω scans	*h* = −15→16
Absorption correction: multi-scan (CrysAlis PRO; Oxford Diffraction, 2010)	*k* = −10→9
*T*_min_ = 0.941, *T*_max_ = 1.000	*l* = −15→15
6112 measured reflections	

### Refinement

**Table:**

Refinement on *F*^2^	Primary atom site location: structure-invariant direct methods
Least-squares matrix: full	Secondary atom site location: difference Fourier map
*R*[*F*^2^ > 2σ(*F*^2^)] = 0.065	Hydrogen site location: inferred from neighbouring sites
*wR*(*F*^2^) = 0.191	H-atom parameters constrained
*S* = 1.05	*w* = 1/[σ^2^(*F*_o_^2^) + (0.0878*P*)^2^ + 0.0871*P*] where *P* = (*F*_o_^2^ + 2*F*_c_^2^)/3
2645 reflections	(Δ/σ)_max_ = 0.001
173 parameters	Δρ_max_ = 0.42 e Å^−^^3^
1 restraint	Δρ_min_ = −0.21 e Å^−^^3^

### Special details

**Table:**

Experimental. *CrysAlis PRO*, Oxford Diffraction Ltd., Version 1.171.34.40 (release 27–08-2010 CrysAlis171. NET) (compiled Aug 27 2010,11:50:40) Empirical absorption correction using spherical harmonics, implemented in SCALE3 ABSPACK scaling algorithm.
Geometry. All e.s.d.'s (except the e.s.d. in the dihedral angle between two l.s. planes) are estimated using the full covariance matrix. The cell e.s.d.'s are taken into account individually in the estimation of e.s.d.'s in distances, angles and torsion angles; correlations between e.s.d.'s in cell parameters are only used when they are defined by crystal symmetry. An approximate (isotropic) treatment of cell e.s.d.'s is used for estimating e.s.d.'s involving l.s. planes.
Refinement. Refinement of *F*^2^ against ALL reflections. The weighted *R*-factor *wR* and goodness of fit *S* are based on *F*^2^, conventional *R*-factors *R* are based on *F*, with *F* set to zero for negative *F*^2^. The threshold expression of *F*^2^ > σ(*F*^2^) is used only for calculating *R*-factors(gt) *etc*. and is not relevant to the choice of reflections for refinement. *R*-factors based on *F*^2^ are statistically about twice as large as those based on *F*, and *R*- factors based on ALL data will be even larger.

### Fractional atomic coordinates and isotropic or equivalent isotropic displacement parameters (Å^2^)

**Table:**

	*x*	*y*	*z*	*U*_iso_*/*U*_eq_	
Cl1	−0.37380 (8)	0.08507 (14)	−0.01623 (10)	0.0948 (5)	
O1	0.06757 (17)	−0.2327 (3)	0.42795 (18)	0.0656 (7)	
O2	0.22097 (18)	−0.0437 (3)	0.57542 (18)	0.0692 (7)	
N1	−0.2183 (2)	−0.0963 (4)	−0.0205 (2)	0.0647 (8)	
C2	−0.1521 (3)	−0.1394 (4)	0.2107 (3)	0.0606 (9)	
H2	−0.1303	−0.1540	0.2887	0.073*	
C3	−0.0976 (2)	−0.2097 (3)	0.1434 (2)	0.0488 (8)	
C4	−0.1347 (3)	−0.1827 (4)	0.0290 (3)	0.0597 (9)	
H4	−0.0987	−0.2282	−0.0173	0.072*	
C5	−0.2373 (3)	−0.0491 (4)	0.1629 (3)	0.0626 (9)	
H5	−0.2745	−0.0006	0.2070	0.075*	
C6	−0.2664 (2)	−0.0322 (4)	0.0476 (3)	0.0585 (9)	
C7	−0.0017 (2)	−0.3089 (4)	0.1925 (3)	0.0588 (9)	
H7A	−0.0165	−0.3841	0.2451	0.071*	
H7B	0.0140	−0.3677	0.1320	0.071*	
N8	0.0890 (2)	−0.2170 (3)	0.2508 (2)	0.0515 (7)	
C9	0.1498 (2)	−0.1287 (4)	0.1924 (2)	0.0552 (8)	
H9A	0.1105	−0.0411	0.1509	0.066*	
H9B	0.1735	−0.1950	0.1406	0.066*	
C10	0.2408 (3)	−0.0717 (4)	0.2892 (3)	0.0537 (8)	
H10A	0.3021	−0.1365	0.2968	0.064*	
H10B	0.2579	0.0370	0.2782	0.064*	
N11	0.20128 (18)	−0.0880 (3)	0.3875 (2)	0.0475 (6)	
C12	0.1124 (2)	−0.1855 (3)	0.3619 (2)	0.0471 (7)	
C13	0.2523 (3)	−0.0357 (4)	0.4938 (3)	0.0538 (8)	
O14	0.34333 (19)	0.0302 (3)	0.4922 (2)	0.0733 (7)	
C15	0.4057 (3)	0.0951 (5)	0.5990 (4)	0.0937 (14)	
H15A	0.3606	0.1450	0.6379	0.112*	
H15B	0.4527	0.1742	0.5850	0.112*	
C16	0.4668 (3)	−0.0327 (6)	0.6709 (4)	0.1119 (17)	
H16A	0.4200	−0.1055	0.6906	0.168*	
H16B	0.5122	0.0120	0.7386	0.168*	
H16C	0.5075	−0.0869	0.6299	0.168*	

### Atomic displacement parameters (Å^2^)

**Table:**

	*U*^11^	*U*^22^	*U*^33^	*U*^12^	*U*^13^	*U*^23^
Cl1	0.0669 (7)	0.1178 (9)	0.0857 (9)	0.0107 (6)	−0.0010 (6)	0.0055 (6)
O1	0.0580 (15)	0.0992 (17)	0.0429 (13)	−0.0119 (13)	0.0197 (11)	0.0150 (12)
O2	0.0746 (17)	0.1006 (17)	0.0353 (13)	−0.0080 (13)	0.0204 (12)	−0.0088 (12)
N1	0.0613 (19)	0.094 (2)	0.0381 (16)	−0.0072 (16)	0.0124 (14)	0.0020 (14)
C2	0.065 (2)	0.088 (2)	0.0290 (17)	−0.0102 (19)	0.0121 (15)	−0.0066 (16)
C3	0.0470 (18)	0.0610 (18)	0.0385 (17)	−0.0136 (15)	0.0122 (14)	−0.0050 (14)
C4	0.062 (2)	0.081 (2)	0.0403 (19)	−0.0085 (19)	0.0225 (17)	−0.0091 (17)
C5	0.057 (2)	0.089 (2)	0.045 (2)	−0.0033 (18)	0.0180 (16)	−0.0080 (18)
C6	0.0461 (19)	0.075 (2)	0.051 (2)	−0.0093 (16)	0.0088 (16)	−0.0010 (17)
C7	0.062 (2)	0.063 (2)	0.050 (2)	−0.0107 (17)	0.0131 (17)	−0.0077 (16)
N8	0.0525 (16)	0.0653 (16)	0.0366 (15)	−0.0100 (13)	0.0122 (12)	−0.0006 (12)
C9	0.059 (2)	0.076 (2)	0.0357 (18)	−0.0088 (16)	0.0213 (15)	−0.0047 (15)
C10	0.0527 (19)	0.076 (2)	0.0366 (17)	−0.0077 (16)	0.0200 (14)	−0.0029 (15)
N11	0.0430 (14)	0.0696 (16)	0.0304 (14)	−0.0031 (12)	0.0115 (11)	0.0012 (11)
C12	0.0425 (17)	0.0619 (19)	0.0367 (17)	0.0062 (14)	0.0113 (14)	0.0110 (14)
C13	0.054 (2)	0.071 (2)	0.0355 (18)	−0.0015 (17)	0.0117 (15)	0.0011 (16)
O14	0.0653 (16)	0.1055 (19)	0.0469 (15)	−0.0273 (14)	0.0124 (12)	−0.0147 (13)
C15	0.085 (3)	0.130 (4)	0.058 (3)	−0.035 (3)	0.006 (2)	−0.020 (3)
C16	0.073 (3)	0.174 (5)	0.075 (3)	−0.019 (3)	0.000 (3)	−0.024 (3)

### Geometric parameters (Å, °)

**Table:**

Cl1—C6	1.743 (3)	N8—C9	1.443 (3)
O1—C12	1.209 (3)	C9—C10	1.522 (4)
O2—C13	1.198 (3)	C9—H9A	0.9700
N1—C6	1.315 (4)	C9—H9B	0.9700
N1—C4	1.333 (4)	C10—N11	1.462 (3)
C2—C5	1.364 (5)	C10—H10A	0.9700
C2—C3	1.387 (4)	C10—H10B	0.9700
C2—H2	0.9300	N11—C13	1.368 (4)
C3—C4	1.375 (4)	N11—C12	1.411 (4)
C3—C7	1.512 (4)	C13—O14	1.347 (4)
C4—H4	0.9300	O14—C15	1.452 (4)
C5—C6	1.371 (5)	C15—C16	1.491 (5)
C5—H5	0.9300	C15—H15A	0.9700
C7—N8	1.451 (4)	C15—H15B	0.9700
C7—H7A	0.9700	C16—H16A	0.9600
C7—H7B	0.9700	C16—H16B	0.9600
N8—C12	1.343 (4)	C16—H16C	0.9600
			
C6—N1—C4	115.8 (3)	H9A—C9—H9B	109.2
C5—C2—C3	120.1 (3)	N11—C10—C9	102.9 (2)
C5—C2—H2	119.9	N11—C10—H10A	111.2
C3—C2—H2	119.9	C9—C10—H10A	111.2
C4—C3—C2	116.4 (3)	N11—C10—H10B	111.2
C4—C3—C7	121.5 (3)	C9—C10—H10B	111.2
C2—C3—C7	122.1 (3)	H10A—C10—H10B	109.1
N1—C4—C3	125.0 (3)	C13—N11—C12	124.4 (2)
N1—C4—H4	117.5	C13—N11—C10	124.3 (2)
C3—C4—H4	117.5	C12—N11—C10	110.6 (2)
C2—C5—C6	117.6 (3)	O1—C12—N8	127.2 (3)
C2—C5—H5	121.2	O1—C12—N11	126.4 (3)
C6—C5—H5	121.2	N8—C12—N11	106.4 (2)
N1—C6—C5	125.0 (3)	O2—C13—O14	124.8 (3)
N1—C6—Cl1	116.1 (3)	O2—C13—N11	126.0 (3)
C5—C6—Cl1	118.9 (3)	O14—C13—N11	109.2 (3)
N8—C7—C3	113.2 (2)	C13—O14—C15	115.8 (3)
N8—C7—H7A	108.9	O14—C15—C16	109.9 (3)
C3—C7—H7A	108.9	O14—C15—H15A	109.7
N8—C7—H7B	108.9	C16—C15—H15A	109.7
C3—C7—H7B	108.9	O14—C15—H15B	109.7
H7A—C7—H7B	107.7	C16—C15—H15B	109.7
C12—N8—C9	113.9 (2)	H15A—C15—H15B	108.2
C12—N8—C7	122.2 (2)	C15—C16—H16A	109.5
C9—N8—C7	123.0 (3)	C15—C16—H16B	109.5
N8—C9—C10	102.3 (2)	H16A—C16—H16B	109.5
N8—C9—H9A	111.3	C15—C16—H16C	109.5
C10—C9—H9A	111.3	H16A—C16—H16C	109.5
N8—C9—H9B	111.3	H16B—C16—H16C	109.5
C10—C9—H9B	111.3		
			
C5—C2—C3—C4	−0.1 (5)	C9—C10—N11—C13	−173.1 (3)
C5—C2—C3—C7	179.1 (3)	C9—C10—N11—C12	16.0 (3)
C6—N1—C4—C3	0.8 (5)	C9—N8—C12—O1	173.2 (3)
C2—C3—C4—N1	−0.6 (5)	C7—N8—C12—O1	4.0 (5)
C7—C3—C4—N1	−179.8 (3)	C9—N8—C12—N11	−7.7 (3)
C3—C2—C5—C6	0.4 (5)	C7—N8—C12—N11	−177.0 (2)
C4—N1—C6—C5	−0.5 (5)	C13—N11—C12—O1	2.1 (5)
C4—N1—C6—Cl1	178.4 (2)	C10—N11—C12—O1	173.0 (3)
C2—C5—C6—N1	−0.1 (5)	C13—N11—C12—N8	−177.0 (3)
C2—C5—C6—Cl1	−179.0 (2)	C10—N11—C12—N8	−6.1 (3)
C4—C3—C7—N8	106.3 (3)	C12—N11—C13—O2	−12.3 (5)
C2—C3—C7—N8	−72.9 (4)	C10—N11—C13—O2	178.0 (3)
C3—C7—N8—C12	91.0 (3)	C12—N11—C13—O14	169.1 (3)
C3—C7—N8—C9	−77.3 (4)	C10—N11—C13—O14	−0.6 (4)
C12—N8—C9—C10	17.3 (3)	O2—C13—O14—C15	−0.1 (5)
C7—N8—C9—C10	−173.5 (3)	N11—C13—O14—C15	178.6 (3)
N8—C9—C10—N11	−18.9 (3)	C13—O14—C15—C16	82.3 (4)

### Hydrogen-bond geometry (Å, °)

**Table:**

*D*—H···*A*	*D*—H	H···*A*	*D*···*A*	*D*—H···*A*
C4—H4···O1^i^	0.93	2.53	3.366 (5)	150
C9—H9B···O2^i^	0.97	2.50	3.395 (4)	152

Symmetry codes: (i) *x*, −*y*−1/2, *z*−1/2.
